# A Comparison of Match Demands Using Ball-In-Play versus Whole Match Data in Professional Soccer Players of the English Championship

**DOI:** 10.3390/sports9060076

**Published:** 2021-05-26

**Authors:** Dylan Mernagh, Anthony Weldon, Josh Wass, John Phillips, Nimai Parmar, Mark Waldron, Anthony Turner

**Affiliations:** 1 Queens Park Rangers Football Club, London W12 7PJ, UK; Dylan.Mernagh@qpr.co.uk; 2 Department of Sports and Recreation, Faculty of Management and Hospitality, The Technological and Higher Education Institute of Hong Kong, Hong Kong, China; 3 Athlete Health Intelligence, English Institute of Sport, Manchester M11 3BS, UK; Joshua.Wass@eis2win.co.uk; 4 FK Krasnodar, 350901 Krasnodar, Russia; johnphillips1981@hotmail.co.uk; 5 Faculty of Science and Technology, London Sports Institute, Middlesex University London, London NW4 4BT, UK; n.parmar@mdx.ac.uk (N.P.); a.n.turner@mdx.ac.uk (A.T.); 6 Applied Sport Technology Exercise and Medicine Research Centre (A-STEM), College of Engineering, Swansea University, Swansea SA1 8EN, UK; mark.waldron@swansea.ac.uk

**Keywords:** high-speed running, acceleration, metabolic load, football, sport

## Abstract

This is the first study to report the whole match, ball-in-play (BiP), ball-out-of-play (BoP), and Max BiP (worst case scenario phases of play) demands of professional soccer players competing in the English Championship. Effective playing time per soccer game is typically <60 min. When the ball is out of play, players spend time repositioning themselves, which is likely less physically demanding. Consequently, reporting whole match demands may under-report the physical requirements of soccer players. Twenty professional soccer players, categorized by position (defenders, midfielders, and forwards), participated in this study. A repeated measures design was used to collect Global Positioning System (GPS) data over eight professional soccer matches in the English Championship. Data were divided into whole match and BiP data, and BiP data were further sub-divided into different time points (30–60 s, 60–90 s, and >90 s), providing peak match demands. Whole match demands recorded were compared to BiP and Max BiP, with BiP data excluding all match stoppages, providing a more precise analysis of match demands. Whole match metrics were significantly lower than BiP metrics (*p* < 0.05), and Max BiP for 30–60 s was significantly higher than periods between 60–90 s and >90 s. No significant differences were found between positions. BiP analysis allows for a more accurate representation of the game and physical demands imposed on professional soccer players. Through having a clearer understanding of maximum game demands in professional soccer, practitioners can design more specific training methods to better prepare players for worst case scenario passages of play.

## 1. Introduction

The game of soccer is acyclical, with the activity and intensity of players’ actions unpredictable [1]. Soccer is characterized by short bouts of high-intensity running, with longer periods of lower intensity activity [2,3,4]. Match outcomes can be determined by explosive actions and high-intensity passages of play, leading to increased assists and goals scored [5]. These instances can differentiate between elite and sub-elite playing levels [6]. Therefore, to optimize soccer players’ training and preparation, and reduce injury risk, comprehensively understanding the physical demands of soccer is imperative. 

Modern microtechnology (i.e., Global Positioning System (GPS) can be used to quantify individual and team training loads and has become commonplace in semi-professional and professional soccer club [7,8,9,10]. GPS microtechnology provides reliable and valid measurements of over-ground speed, from which several kinematic variables can be derived [11,12], including distance and relative distance in selected speed zones, and the frequency of accelerations and decelerations [13]. Internal loading measures, such as heart rate, are also commonly reported alongside external load variables [14].

Most soccer studies have reported whole or part match GPS-derived data [15], showing that distances covered at various intensities differ between positional groups [16]. Current game demands show that midfield players cover the most total distance [15,17,18], whilst wide players and forwards complete more high-intensity running [17,19]. Furthermore, it has been reported during English Premier League matches, players stood for 5.6% of total time, walked (0.7–7.1 km·h^−1^) for 60%, jogged (7.2–14.3 km·h^−1^) 26%, and performed running (14.4–19.7 km·h^−1^) for 6%, high speed running (19.8–25.1 km·h^−1^) for 2%, and sprinting (>25.1 km·h^−1^) for 1% [19]. Although this provides useful information about the volume of activity, it does not accurately reflect fluctuations in physical, technical, or tactical intensity. Subsequently, this underestimates the most intense periods of match play [10]. Such analysis may lead to players being underprepared for the most demanding and crucial moments of competitive soccer matches.

Research has attempted to quantify match demands, with 5-min rolling averages [10], but this method only accounts for broad fluctuations in intensity; it does not consider periods of reduced intensity, such as when the ball is out of play (BoP), which has been investigated in other football codes [20]. This could mean 5-min rolling averages also underestimate the physical demands. For example, the concept of effective playing time was analyzed at a men’s European Soccer Championship, which showed the game was active for 54.4 ± 4 min [21]. More recently, match activity profiles during periods of ball-in-play (BiP) were reported to provide a more accurate representation of match demands compared to whole match variables [8,9,22]. This concept aims to exclude all stoppages in play from the analysis, such as substitutions, the ball leaving the field of play, and dead play during free kicks or set pieces. All GPS metrics during BiP periods were significantly higher than whole match averages, thus providing a detailed insight of match running demands and informing subsequent training requirements. The authors also isolated set passages of BiP into 30–60 s, 60–90 s, and >90 s periods, to understand the relationship between running intensity and the duration of BiP periods. Furthermore, investigation of maximum outputs for each GPS metric (rather than the average) was thought to reveal peak demands of match play, otherwise known as worst case scenarios [23]. In support of this reasoning, there were higher values for peak demands compared to average, with the highest values attained during the shortest periods [9]. It was suggested that if the physical capacity of a player is not sufficient to cope with these demands, then performance is likely to suffer. It is vital, however, that this association is now assessed at the senior level of the professional game, to ensure the training methods used induce an appropriate match-related stimulus. We propose that by understanding game demands using BiP metrics, practitioners can better appreciate work:rest ratios during BiP periods and how they change based on the time period of play. Furthermore, such data will be valuable for practitioners when designing and periodizing individual and team practices.

Therefore, this study aimed to investigate the Whole Match, Mean BiP, Mean BoP, and Max BiP match demands using common GPS metrics and set passages of play, in professional soccer players, as well as across different positions (defenders, midfielders, and forwards). It was hypothesized that Mean BiP and Max BiP demands would be significantly higher across all GPS metrics in comparison to whole match demands. Furthermore, it was theorized that peak match demands would be highest over the shortest passages of play, and that midfielders would record the highest values.

## 2. Materials and Methods

### 2.1. Participants

A total of 20 professional male soccer players of the English Championship (age: 24 ± 4 years; height: 180.8 ± 8.0 cm; body mass: 80.7 ± 10.3 kg) participated in this study. Before providing written consent, all players were given an outline of the study’s rationale and procedures. All players were healthy, undertaking full training, and were familiar with wearing GPS units due to it being part of their routine monitoring procedures. Data were included if players had played ≥60 min, because substitutions have shown to produce relatively higher physical outputs, which may skew results [23]. Ethical approval was granted by an institutional committee with the study conforming to the recommendations of the Declaration of Helsinki.

### 2.2. Observational Study Design

Commonly reported GPS metrics (Table 1) were compared based on conventional whole match, BiP, and Max BiP analysis (with the latter representing peak match demands in play). Participants were categorized into positional groups (defenders, midfielders, and forwards) to assess differences in positional demands. Given the typical inverse relationship noted between volume and intensity, Max BiP periods were split into 30–60 s, 60–90 s, and >90 s time periods.

Data were collected over eight competitive matches of the English Championship between February and May 2018. The 18 Hz GPS units (APEX Pod, STATSports, Belfast, UK) were placed in bespoke pockets in the players’ match shirts, between their shoulder blades close to their thoracic spine, thus minimizing movement artifacts [24]. A timeline of the duration of all plays was generated by SportsCode (SportsCode, Sportstec, Lower Hutt, New Zealand) to define BiP, BoP, and Max BiP. The duration in which play is ongoing before the ball exited the pitch or the referee stopped play was considered as BiP. In contrast, BoP is the duration in which the play ceases due to the ball exiting the pitch or the referee stopping play, and before the play resumes. Max BiP represents the most physically demanding period (peak match demand) and is the maximum output occurring during a BiP period >30 s. As it was hypothesized that Max BiP is dependent on the duration of BiP, peak match demand phases of play were split into 30–60 s, 60–90 s, and >90 s time periods [9,25]. To limit the possibility of inaccurate data, short plays (i.e., <30 s) with high bouts of intensity were not included.

Data were downloaded using the appropriate software (APEX PSA Software, Version 2.6.1.176, STATSports, Belfast, UK) and time periods were split manually for the whole match period following video playback. SportsCode generated a timeline of the game (SportsCode, Sportstec, Lower Hutt, New Zealand) and was then integrated into the software to automatically split the match data into periods of BiP, BoP, and Max BiP. Data were then exported into Microsoft Excel (Microsoft Corporation, Redmond, WA, USA) to transfer the SportsCode-generated timeline into BiP durations. A data workflow was then created in Alteryx Designer 11.7, (Alteryx, Irvine, CA, USA) to ascertain periods of BoP and Max BiP.

### 2.3. Statistical Analyses

SPSS version 24 was used to run a two-way 3 (playing position: defenders, midfielders, and forwards) × 3 (analysis type: Whole Match, BiP, BoP, and Max BiP) mixed ANOVA with repeated measures on GPS metric type (see Table 1). This analysis enabled statistical differences in the dependent variable (analysis type) to be determined, as well as any interaction effects with the independent variable (playing position) to be noted. Where sphericity was violated, the Greenhouse–Geisser correction was used, and Bonferroni adjustment was used for post hoc analysis. Significance was set as *p* < 0.05 and effect sizes were calculated using partial η^2^. Normality was confirmed using the Shapiro–Wilk test.

## 3. Results

Differences were found across all metrics when comparing across Whole Match, Mean BiP, Mean BoP, Max BiP, and each individual position. Comparisons are reported in Table 2, with values as follows: mean meters per minute (F (3, 51) = 1342.7; *p* < 0.01; partial η^2^ = 0.987), mean HSR per minute (F (3, 51) = 588.48; *p* < 0.01; partial η^2^ = 0.972), mean accelerations per minute (F (3, 51) = 1102.32; *p* < 0.01; partial η^2^ = 0.985), mean decelerations per minute (F (3, 51) = 1035.9; *p* < 0.01; partial η^2^ = 0.984), and mean HMLD per minute (F (2, 34) = 603.23; *p* < 0.01; partial η^2^ = 0.973).

When comparing across durations, differences were found across all metrics. There were positional differences for Whole Match meters, BiP, BoP, and Max BiP (see Table 2). Comparisons are reported in Table 3, with values as follows: Max BiP meters per minute (F (2, 34) = 277.57; *p* < 0.01; partial η^2^ = 0.942), HSR (F (2, 34) = 162.24; *p* < 0.01; partial η^2^ = 0.908), Max BiP accelerations per minute (F (2, 34) = 272.68; *p* < 0.01; partial η^2^ = 0.941), Max BiP decelerations per minute (F (2, 48) = 63.68 *p* < 0.01; partial η^2^ = 0.726), and Max BiP HMLD per minute (F (2, 48) = 92.66; *p* < 0.01; partial η^2^ = 0.794).

## 4. Discussion

This study aimed to analyze Whole Match, Mean and Max BiP demands, and Mean BoP for professional soccer players competing in the English Championship. Results revealed significant differences across all metrics (meters per minute, HSR per minute, accelerations per minute, decelerations per minute, and HML per minute) for Whole Match, Mean BiP, Mean BoP, and Max BiP. As hypothesized, the metrics for Mean BiP were significantly higher than the metrics for mean Whole Match data and the metrics for Max BiP were significantly higher than for Mean BiP (Table 2). There were also several significant differences reported between positions for meters per minute, accelerations per minute, and HML per minute, but not for HSR per minute (Table 2). Results further revealed that Max BiP is time-dependent, as Max BiP periods between 30 and 60 s were significantly higher than Max BiP periods between 60–90 s and >90 s (Table 3).

Therefore, to quantify peak match demands in soccer, it is important to identify the most appropriate method of analysis [10]. This study differs from previous match analysis in football where whole match, part match, segmental, and rolling average analyses have been used to quantify workloads [26]. Measures such as the whole match and part match values can be limited in use as they provide an absolute measure of the physical demands of competition. Moving averages have been used in research as the optimal measure of peak match demands [10]. However, BiP analysis is relatively new and, as such, there is a paucity of research in this area. Subsequently, the results of this study show that whole match data (which include periods when the ball is out of play) underestimate movement demands and support the growing research in this area [8,9,23,25]. These findings also support the research of Riboli et al. [8] in Italian Serie A soccer players, who state discrete time frames, such as the most demanding periods of play, should be considered to properly condition players. Furthermore, Riboli et al. [8] state that failure to do so may underestimate the outputs required when only considering whole match data samples.

This study demonstrates that peak match demands are time-dependent, as Max BiP demands for 30–60 s are significantly higher from Max BiP periods for 60–90 s, and >90 s. When using time-dependent Max BiP periods, it is important to have a minimum duration threshold, which ensures very short plays with high bouts of intensity are not included, to reduce the likelihood of producing inaccurate data [25]. Conversely, the rolling average analysis method, which typically uses a broader time period of 5-min [3], may not be discrete enough to isolate short passages of high workload. Data in a 5-min period may provide a suboptimal measure of peak match demands, and lead to an underestimation when compared to our results. For example, peak running demands using 5-min rolling average range from 129 to 148 m/min, but using Max BiP, we report ranges from 135 to 210 m/min, depending on time and position.

This analysis highlights periods of high-intensity work, which may not be recognized in a whole or part match analysis but allow for a more specific prescription in the training microcycle through intensity and individuality. Max BiP total distances show a decline over 30–60 s (195 m/min), 60–90 s (156 m/min) and >90 s (143 m/min). The results show that BiP data are time-dependent and BiP values will naturally decrease over longer periods of time due to the physiological, contextual, and technical/tactical demands of the sport [8,10,25]. Practitioners can use the Mean and Max BiP (peak match demands) to align or supersede training metrics to help coaches prepare soccer players for the specific demands of the game and worse case scenarios [27]. Furthermore, exposing players to high match-play demands including high-speed running may also reduce the likelihood of injuries to the lower limbs, such as the hamstrings [27].

An interesting finding of this study is that midfield players completed higher amounts of HSR across the Whole Match, BiP, and Max BiP. Previous research in elite Spanish [28] and Italian soccer [8] found that using absolute GPS match data, wide midfielders completed significantly more HSR than central midfielders. Furthermore, forwards and full-backs completed more HSR than central midfield players, but these values were not statistically significant [28]. The results of this study are supported by numerous studies using absolute HSR values [17,19]. It is likely that the principal difference in results found within this study and others is the use of BiP analysis. This is further demonstrated as forward players cover more HSR during BoP periods, potentially due to the need to reposition quickly to prevent opposition attacks when out of possession or conversely, to gain an advantageous position when in possession. Our data therefore suggest that BiP could offer fresh insight into the positional match demands of professional soccer players.

It has been suggested that for the field of sports science to progress within soccer and the application of physical match data, practitioners must compare and contrast methodologies that develop an understanding of contextualizing game demands [29]. Therefore, future research can include tactical factors such as formation, with researchers and practitioners advised to consider these factors when comparing the results of different studies within the literature.

## 5. Limitations

Given that this study was conducted in one professional team, it was not possible to increase the sample size to enable an analysis of position beyond the broad grouping of defenders, midfielders, and forwards. This also meant that wide midfielders and central midfielders were grouped together, which may affect results. However, to enable a more in-depth analysis such as this (i.e., using standardized GPS-based microtechnology) across homogenous teams (e.g., professional, same league), future research is likely to require the collaboration of several soccer clubs and sport science departments. Furthermore, tactical formation has demonstrated a significant effect on high-intensity running for forward players [19] and may further explain why our results do not compare with the results of other studies. The playing formation in the current study was 4-4-2; however, this was subject to tactical changes within the game, which may have influenced physical output data [19]. This is challenging for researchers as there is more fluidity in tactical formations in modern football [30].

## 6. Practical Applications

The challenge within soccer is the inclusion of other aspects of performance that occur simultaneously during match performance. These physical demands happen concurrently alongside technical and tactical aspects within a match context. By gaining a greater understanding of the typical and maximum demands of duration specific movement in professional soccer players, training can be designed to match, or supersede, these metrics, whilst being monitored by GPS for feedback. This method may allow for greater specificity and transfer to performance in match play. This also considers the different physical requirements of defenders, midfielders, and forwards. Coupling this with the increased synchronicity of the technical and tactical demands allows players to execute skills and decision making above game speed, which should ultimately aid performance.

## 7. Conclusions

This study is the first to report the whole match, BiP, BoP, and max BiP demands of professional soccer players competing in the English Championship. Using BiP analysis allows for an accurate representation of game demands, providing a more comprehensive understanding of the physical demands imposed on professional soccer players. This in turn allows practitioners to effectively program training to achieve a conditioning stimulus representative of the speed of the game. The normative data herein act as a guide to drive the intensity of training, noting that for all metrics, intensity is time period sensitive, and for some metrics, it is also position-specific. These key details can be used to shape training design and provisions around work:rest periods for practices and games.

## Figures and Tables

**Table 1:** Analyzed metrics and operational descriptions from the GPS units.

Metric	Description
Meters Per Minute (m/min)	Total distance covered (m)/Total minutes (min)
High Metabolic Load Distance Per Minute (HMLD/min)	Distance accelerating over 2.5 m·s^−2^ and sprinting over 5.5 m·s^−1^/Total minutes
High Speed Running Per Minute (HSR/min)	Distance covered over 5m·s^−2^/Total minutes
Accelerations Per Minute (Acc/min)	Change in velocity over 3 m·s^−2^/Total minutes
Decelerations Per Minute (Dec/min)	Change in velocity over 3 m·s^−2^/Total minutes

**Table 2:** Comparison of Whole Match, Mean BiP, Mean BoP, and Max BiP, across positions.

Metric	Position	Whole Match	Ball in Play (BIP)	Ball out Play (BOP)	Max BIP
Meters Per Minute (m/min)	Defender	92.9 ± 6.6 _xyzβ_	118.2 ± 11.4 _wyzµ_	19.5 ± 7.2 _wxzµβ_	154.9 ± 12.5 _wxyµ_
	Midfield	96.6 ± 10.2 _xyzβ_	140 ± 11.5 _wyz α_	31.2 ± 5.8 _wxzα_	179.3 ± 9.1 _wxyα_
	Forward	73.9 ± 9.7 _xyzαµ_	125.4 ± 15.7 _wyz_	41.1 ± 9.3 _wxzα_	161 ± 15.2 _wxy_
	Total	88.5 ± 13	128 ± 15.4	30 ± 11.4	165.3 ± 16
High Speed Running Per Minute (HSR/min)	Defender	8.7 ± 1.8 _xyz_	13.7 ± 4.2 _wyz_	3.2 ± 1.8 _wxz_	41.5 ± 7.6 _wxy_
	Midfield	9.6 ± 1.1 _xyz_	18.7 ± 2.8 _wyz_	4.9 ± 1.3 _wxz_	49 ± 7.4 _wxy_
	Forward	8.2 ± 2.7 _xyz_	18.5 ± 5.2 _wyz_	5.7 ± 2.7 _wxz_	48.2 ± 9.4 _wxy_
	Total	8.6 ± 1.9	16.9 ± 4.6	4.5 ± 2.1	46.1 ± 8.4
Accelerations Per Minute (Acc/min)	Defender	1.1 ± 0.1 _xyzβ_	1.7 ± 0.2 _wyzµ_	0.5 ± 0.1 _wxzβ_	3.7 ± 0.1 _wxy_
	Midfield	1.2 ± 0.2 _xyzβ_	2.0 ± 0.2 _wyzαβ_	0.6 ± 0.1 _wxz_	4.1 ± 0.3 _wxy_
	Forward	0.9 ± 0.1 _xyzαµ_	1.6 ± 0.3 _wyzµ_	0.8 ± 0.3 _wxzα_	3.7 ± 0.5 _wxy_
	Total	1.1 ± 0.2	1.8 ± 0.3	0.6 ± 0.2	3.8 ± 0.4
Decelerations Per Minute (Dec/min)	Defender	1.0 ± 0.1 _xyzβ_	1.5 ± 0.2 _wyzµ_	0.5 ± 0.1 _wxz_	3.5 ± 0.4 _wxy_
	Midfield	1.0 ± 0.1 _xyzβ_	1.8 ± 0.2 _wyzαβ_	0.6 ± 0.2 _wxz_	3.8 ± 0.1 _wxyβ_
	Forward	0.7 ± 0.1 _xyzαµ_	1.5 ± 0.2 _wyzµ_	0.6 ± 0.2 _wxz_	3.3 ± 0.2 _wxyµ_
	Total	0.9 ± 0.2	1.6 ± 0.2	0.6 ± 0.2	3.6 ± 0.3
High Metabolic Load Distance Per Minute (HMLD/min)	Defender	16.1 ± 2.3 _xyz_	25.3 ± 5.2 _wyzµ_	7 ± 2.9 _wxzβ_	50.6 ± 8.9 _wxy_
	Midfield	18.5 ± 2.1 _xyzβ_	34.2 ± 3.8 _wyzα_	9.8 ± 1.5 _wxzα_	62.3 ± 6.6 _wxy_
	Forward	13.8 ± 3.2 _xyzµ_	29.5 ± 6.1 _wyz_	11 ± 3.2 _wxz_	56.2 ± 10.4 _wxy_
	Total	16.3 ± 3.1	29.7 ± 6.1	9.2 ± 3.0	56.4 ± 9.6

Key: w = Significantly different to Whole Match, x = Significantly different to BiP, y = Significantly different to BoP, z = Significantly different to Max BiP, α = Significantly different to Defender, β = Significantly different to forward, µ = Significantly different to midfield.

**Table 3:** Comparison of Max BiP metrics, based on duration and playing position.

Metric	Position	Max for Plays 30–60 S	Max for Plays 60–90 S	Max for Plays >90 S
Meters Per Minute (m/min)	Defender	183.5 ± 15.7 _yzµ_	144.1 ± 13.9 _xzµ_	136.9 ± 10.6 _xyµ_
	Midfield	210 ± 9.7 _yzαβ_	170.9 ± 8.5 _xzα_	157.1 ± 10.9 _xyα_
	Forward	193.7 ± 21.6 _yxµ_	154.1 ± 16.7 _xz_	135.3 ± 13 _xy_
	Total	195.8 ± 19	156.5 ± 17.1	143.5 ± 14.9
High Speed Running Per Minute (HSR/min)	Defender	69.3 ± 14.8 _yz_	35.5 ± 9.9 _xz_	19.8 ± 6.1 _yz_
	Midfield	84.2 ± 15.7 _yz_	38.3 ± 5.9 _xz_	24.1 ± 6.5 _yz_
	Forward	75.6 ± 16.5 _yz_	44 ± 9.5 _xz_	25.2 ± 10.4 _yz_
	Total	76.5 ± 16.1	39 ± 16.1	22.9 ± 7.7
Accelerations Per Minute (Acc/min)	Defender	5.7 ± 0.5 _yz_	3 ± 0.4 _z_	2.5 ± 0.4 _xy_
	Midfield	5.9 ± 0.5 _yz_	3.4 ± 0.3 _x_	3 ± 0.5 _xy_
	Forward	5.6 ± 0.8 _yz_	3 ± 0.4 _xz_	2.4 ± 0.5 _xy_
	Total	5.8 ± 0.6	3.1 ± 0.4	2.6 ± 0.6
Decelerations Per Minute (Dec/min)	Defender	5.0 ± 0.9 _yz_	3.0 ± 0.6 _x_	2.5 ± 0.4 _x_
	Midfield	5.8 ± 0.6 _yz_	3.2 ± 0.4 _xz_	2.6 ± 0.5 _x_
	Forward	5.3 ±1.3 _yz_	2.5 ± 0.4 _x_	2.7 ± 0.8 _x_
	Total	5.4 ± 1.0	2.9 ± 0.5	2.4 ± 0.6
High Metabolic Load Distance Per Minute (HMLD/min)	Defender	76.4 ± 14.9 _yz_	43 ± 9.4 _xz_	32.2 ± 6.4 _xy_
	Midfield	91.6 ± 12 _yz_	53.6 ± 5.8 _xz_	41. 8 ± 6.8 _xy_
	Forward	81.7 ± 18.3 _yz_	51.8 ± 9.3 _xz_	35.1 ± 10.1 _xy_
	Total	83.3 ± 15.7	49.5 ± 9.3	36.4 ± 8.5

Key: x = Significantly different to Max for plays 30–60 s, y = Significantly different to Max for plays 60–90 s, z = Significantly different to Max for plays >90 s, α = Significantly different to Defender, β = Significantly different to forward, µ = Significantly different to midfield.

## Data Availability

Data sharing does not apply to this article.
